# Sex disparity in laparoscopic bariatric surgery outcomes: a matched-pair cohort analysis

**DOI:** 10.1038/s41598-021-92254-4

**Published:** 2021-06-17

**Authors:** Pouria Mousapour, Erfan Tasdighi, Alireza Khalaj, Maryam Mahdavi, Majid Valizadeh, Hamidreza Taheri, Farhad Hosseinpanah, Maryam Barzin

**Affiliations:** 1 Obesity Research Center, Research Institute for Endocrine Sciences, Shahid Beheshti University of Medical Sciences, Tehran, Iran; 2 Tehran Obesity Treatment Center, Department of Surgery, Faculty of Medicine, Shahed University, Tehran, Iran

**Keywords:** Weight management, Risk factors

## Abstract

Men have been historically considered to be higher-risk patients for bariatric surgery compared to women, the perception of which is suggested to be a barrier to bariatric surgery in men. The purpose of this study is to conduct a matched-pair analysis to evaluate sex disparities in laparoscopic bariatric surgery outcomes. Data on patients who underwent laparoscopic bariatric surgery from March 2013 to 2017 was collected prospectively. Then, 707 men and 707 women pair-matched for age, preoperative body mass index (BMI) and the procedure type (i.e., sleeve gastrectomy, Roux-en-Y, or one-anastomosis gastric bypass) were compared in terms of weight loss, remission of obesity-related comorbidities, and postoperative complications classified according to the Clavien–Dindo classification. There was no difference between the two sexes regarding the operation time, bleeding during surgery and length of postoperative hospital stay. We observed similar total weight loss, BMI loss, and percentage of excess BMI loss at 12, 24, and 36 months postoperatively between men and women, with no difference in remission of diabetes mellitus, hypertension and dyslipidemia at 12 months. The rate of in-hospital, 30-day and late complications according to Clavien–Dindo classification grades was similar between men and women. Our matched-pair cohort analysis demonstrated that bariatric surgery results in comparable short- and mid-term efficacy in men and women, and is associated with similar rate and severity of postoperative complications between sexes. These findings suggest bariatric surgeons not to consider sex for patient selection in bariatric surgery.

## Introduction

Severe obesity is an enormous health challenge with an exponential increase in its global prevalence over the past several decades^1,2^. Severe obesity and its related chronic conditions such as diabetes mellitus, dyslipidemia, hypertension, and cardiovascular diseases contribute to a dramatic decrease in the quality of life and longevity of individuals^3,4^. Bariatric surgery has long been recognized as the most efficacious treatment for severe obesity and its comorbidities, with postoperative mortality rates decreased to less than 0.1% over the past decade^5^. The global number of patients undergoing bariatric surgery is steadily increasing due to the rise in demand and accessibility and advancements in laparoscopic approaches^6^.

Data indicates that women are more frequently involved in surgical weight loss interventions than men are^7–9^, and according to the latest global reports, over 70% of patients undergoing bariatric surgery are women^10^. Although the underlying reasons for this sexual disparity are not clearly understood, not only can greater body image dissatisfaction and psychological disturbances in women result in greater use of weight loss procedures^11,12^, limited knowledge on postoperative outcomes and surgical complications may also act as meaningful barriers to provide bariatric surgery to men. Deeper insight into the efficacy of this treatment method and its modes of failure could provide surgeons support for decisions on patient selection and improving surgical outcomes.

The purpose of this study is to conduct a matched-pair analysis to evaluate the independent impact of sex on the outcomes of bariatric surgery in terms of weight loss, remission of comorbidities, and incidence of surgical complications.

## Materials and methods

### Patient selection and matching criteria

Tehran Obesity Treatment Study (TOTS) is a prospective study of patients referred to our specialized center to undergo bariatric surgery. Patients aged 18–65 years with body mass index (BMI) ≥ 40 kg/m^2^ or between 35 and 40 kg/m^2^ in the presence of at least one obesity-related comorbidity were enrolled in this study after providing written informed consent. A detailed description of the TOTS is provided elsewhere^13^. A total of 2542 females and 707 males underwent primary bariatric surgery from March 2013 to March 2017 and had completed 36-months follow-up data were enrolled. Men and women were pair-matched at baseline regarding age (± 8 years), preoperative BMI (± 0.6 kg/m^2^), and the procedure type (i.e., sleeve gastrectomy, Roux-en-Y or one-anastomosis gastric bypass). The rates of 12-months, 24-months, and 36-months follow-up were 92.6, 83.5, and 78.8% for men and 94.4, 88.1 and 81.1% for women, respectively.

### Anthropometrics and laboratory measurements

Demographic characteristics were recorded, and anthropometric and biochemical assessments were performed before surgery and during scheduled postoperative visits according to the study protocol^13^.

Body composition was evaluated using the portable bioelectrical impedance analyzer (InBody 370, Biospace, Seoul, Korea). Participants were asked to comply with the following criteria prior to impedance analysis: fasting overnight or for a minimum of 4–5 h, no exercise for at least 12 h, no alcohol consumption for at least 24 h, balanced hydration, and lying in supine position for at least 5 min prior to examination. Resistance to alternating current flow (500- μA at 50/60 kHz) was measured with the patient standing on the analyzer’s platform and interpreted using the “standard” option of the manufacturer’s software.

### Surgical procedures

After a multidisciplinary evaluation of each patient, the surgeon provided the patients a thorough explanation regarding surgical methods, outcomes, and possible complications. All the procedures were completed laparoscopically and under general anesthesia by a single surgical team at three university hospitals. The patients underwent either laparoscopic sleeve gastrectomy (SG), Roux-en-Y gastric bypass (RYGB), or one-anastomosis gastric bypass (OAGB) as described previously by the authors^13^. SG was performed by creating a gastric tube over a 36-F bougie with the exclusion of 80% of the stomach. For RYGB, a vertical stomach pouch with anastomosis to a 150-cm roux jejunum limb was constructed and a side-to-side jejunojejunostomy with a 50-cm biliopancreatic limb was created. OAGB was performed using a 160-cm biliopancreatic limb.

### Follow-up and postoperative care

After discharge, all the patients were started on a liquid diet for two weeks followed by a semi-solid diet for four weeks before resuming a normal diet. All the patients received daily oral multivitamin and mineral supplementations during the first postoperative year as follows: one Pharmaton® capsule daily (Boehringer Ingelheim Inc., Ingelheim am Rhein, Germany, containing 2 mg copper, 10 mg ferrous sulfate, 100 mg folic acid, 1 mcg vitamin B12, vitamins A, B group, C, D, and E, nicotinamide, and biotin) and one Calcicare tablet daily (200 IU vitamin D, 400 mg calcium, 100 mg magnesium, and 4 mg zinc). Optimization of medical therapy for any related comorbidities was provided individually according to our endocrinologist’s recommendations.

Each patient underwent comprehensive assessments by the postoperative care team including an obesity expert, a nutritionist, and an exercise medicine physician at 1, 3, 6, and 12 months, and then annually. Both groups received a similar calorie-restricted diet (with 30–35% protein) and were prescribed daily vitamin and mineral supplements up to 12 months postoperatively. Moreover, all the patients followed a physical activity program (combined aerobic-resistive activity at least 30 min per day) postoperatively.

### Definition of terms

Diabetes mellitus was defined as glycated hemoglobin (HbA1c) ≥ 6.5%, fasting plasma glucose (FPG) ≥ 126 mg/dL, or use of antidiabetic medications^18^. Hypertension was considered as a systolic blood pressure of ≥ 140 mmHg, a diastolic blood pressure of ≥ 90 mmHg, previous diagnosis of hypertension, or use of antihypertensive medication^19^. Dyslipidemia was described as serum triglycerides (TG) ≥ 200 mg/dL, total cholesterol ≥ 240 mg/dL, low-density lipoprotein (LDL) ≥ 160 mg/dL, high-density lipoprotein < 40 mg/dL, or receiving lipid modifying therapy^20^.

Weight reduction outcomes were expressed in percentage of total weight loss (%TWL), BMI loss (BMIL), and percentage of excess BMI loss (%EBMIL) at 12, 24, and 36 months after surgery as:

- TWL% = ([Preoperative weight – Postoperative weight]/ Preoperative weight) × 100.
- BMIL = Preoperative BMI – Postoperative BMI.
- EBMIL% = (BMIL/ [Preoperative BMI − 25]) × 100.

### Statistical analysis

The analyses were performed using SPSS version 22.0 (SPSS Inc., Chicago, IL, USA). Continuous and categorical variables were expressed as mean ± standard deviation (SD) and frequency (percentages), respectively. Categorical, normally distributed, and skewed continuous variables were compared between men and women using Chi-squared test, independent samples *t*-test, and Mann–Whitney U test, respectively. One-way analysis of covariance was used for the comparison of TWL%, BMIL, EBMIL%, and Δ Fat mass between the two sexes with adjustment for preoperative diabetes mellitus, hypertension, and dyslipidemia. Fisher’s exact test was used for the comparison of postoperative complications. The remission of obesity-related comorbidities at one year postoperatively was assessed by unadjusted conditional logistic regression analysis. A *P*-value of less than 0.05 was considered statistically significant.

### Ethical approval and consent to participate

All procedures performed in studies involving human participants were approved and performed in accordance with the ethical standards of the Ethics Committee of the Research Institute for Endocrine Sciences (RIES) of Shahid Beheshti University of Medical Sciences (No. 2ECRIES 93/03/13), with the 1964 Helsinki declaration and its later amendments or comparable ethical standards. Written informed consent was obtained from all individual participants included in the study.

## Results

Of the 3249 patients of the TOTS cohort during 2013–2017, 707 (21.8%) were men. Men were younger and had greater preoperative BMIs in comparison with women. SG was more frequently performed in men than in women. There was no significant difference in the prevalence and the duration of diabetes mellitus, prevalence of insulin therapy, FPG, and HbA1C levels between men and women, preoperatively. The baseline prevalence rates of hypertension and dyslipidemia were higher in men than in women. Men also had greater SBP, DBP, and plasma TG and lower HDL levels than women (Table 1).

**Table 1:** Baseline characteristics of males and females, Tehran Obesity Treatment Study cohort, 2013 to 2019.

Variables	Women	Men	*P*-value
	(n = 2542)	(n = 707)	
Age, years	39.5 ± 11.6	35.8 ± 10.8	< 0.001
BMI, kg/m^2^	44.7 ± 5.8	45.7 ± 6.2	< 0.001
**BMI subgroups, n (%)**			0.013
35–40 kg/m^2^	449 (17.7)	102 (14.4)	
40–50 kg/m^2^	1668 (65.6)	458 (64.8)	
≥ 50 kg/m^2^	425 (16.7)	147 (20.8)	
Weight, kg	115.6 ± 16.3	141.2 ± 21.8	< 0.001
Fat mass, kg	59.1 ± 10.5	63.7 ± 14.6	< 0.001
Fat mass , %	51.1 ± 3.6	44.9 ± 5.9	< 0.001
**Type of procedure, n (%)**			< 0.001
SG	1616 (63.5)	521 (73.7)	
RYGB	126 (5.0)	32 (4.5)	
OAGB	800 (31.5)	154 (21.8)	
**Diabetes mellitus, n (%)**	695 (30.9)	183 (30.4)	0.842
Duration, years	4.0 (1.0 − 10.0)	3.0 (1.0 − 7.0)	0.206
On insulin	137 (5.4)	33 (4.7)	0.504
FPG, mg/dL	109.8 ± 38.3	110.1 ± 35.4	0.849
HbA1c^a^, %	5.6 (5.2–6.1)	5.6 (5.1–6.2)	0.490
Hypertension, n (%)	721 (28.9)	243 (35.5)	0.001
SBP, mmHg	122.9 ± 12.8	125.7 ± 12.7	< 0.001
DBP, mmHg	79.1 ± 8.0	80.7 ± 8.8	< 0.001
Dyslipidemia, n (%)	1299 (53.2)	461 (68.3)	< 0.001
Triglycerides^a^, mg/dL	138.0 (102.0 − 183.0)	154.0 (115.0 − 219.7)	< 0.001
Total cholesterol, mg/dL	189.8 ± 37.2	186.9 ± 40.5	0.078
Cholesterol -LDL, mg/dL	111.2 ± 31.4	109.99 ± 32.96	0.383
Cholesterol -HDL, mg/dL	48.2 ± 11.5	42.2 ± 10.1	< 0.001

BMI, body mass index; SG, sleeve gastrectomy; RYGB, roux-en-y gastric bypass; OAGB, one-anastomosis gastric bypass; FPG, fasting plasma glucose; HbA1c, glycated hemoglobin. SBP, systolic blood pressure; DBP, diastolic blood pressure;

Continuous and categorical variables are represented as mean ± SD and frequency (percentage), respectively.

^a^HbA1c and triglycerides are reported as median (IQR 25–75).

The total of 707 women were pair-matched to 707 men for age (36.7 ± 10.4 versus 35.8 ± 10.8 years), preoperative BMI (45.7 ± 6.2 versus 45.7 ± 6.2 kg/m^2^), and the procedure type (73.7% SG). Their baseline characteristics are reported in Table 2. The matched groups were comparable regarding the prevalence of diabetes mellitus and insulin therapy. Men, however, showed a higher prevalence of hypertension and dyslipidemia than women did.

**Table 2:** Preoperative and operative data of men and women matched for age, preoperative BMI and the type of procedure.

Variables	Women	Men	*P*-value
	(n = 707)	(n = 707)	
Age, years	36.7 ± 10.4	35.8 ± 10.8	0.118
BMI, kg/m^2^	45.7 ± 6.2	45.7 ± 6.2	0.935
Weight, kg	118.8 ± 18.1	141.2 ± 21.8	< 0.001
Fat mass, kg	60.9 ± 11.3	63.7 ± 14.6	< 0.001
Fat mass , %	47.4 ± 14.0	41.7 ± 12.9	< 0.001
**Diabetes mellitus, n (%)**	162 (26.3)	183 (30.4)	0.116
On insulin	24 (3.4)	33 (4.7)	0.224
Hypertension, n (%)	168 (24.1)	243 (35.5)	< 0.001
Dyslipidemia, n (%)	349 (50.9)	461 (68.3)	< 0.001
**Procedure type, n (%)**			0.991
SG	521 (73.7)	521 (73.7)	
RYGB	33 (4.7)	32 (4.5)	
OAGB	153 (21.6)	154 (21.8)	
Operative time, minutes	113.5 ± 27	109.3 ± 26.9	0.007
Intraoperative bleeding, n	6 (0.8)	13 (1.8)	0.106
Length of hospital stay, days	2.2 ± 0.6	2.3 ± 1.4	0.196

BMI, body mass index; SG, sleeve gastrectomy; RYGB, roux-en-y gastric bypass; OAGB, one-anastomosis gastric bypass.

Continuous and categorical variables are represented as mean ± SD and frequency (percentage), respectively.

There was no significant sex disparity in TWL%, BMIL, and EBMIL% at 12, 24 and 36 months postoperatively in both unadjusted and multivariable adjusted analyses. According to BIA analysis, ΔFM was observed to be greater in men compared to women at 12 and 24 months, but this difference was not significant at 36 months postoperatively (Table 3).

**Table 3:** Matched comparison of anthropometric parameters between men and women after 12, 24 and 36 months postoperatively.

	Unadjusted			Adjusted*		
	Women (n = 707)	Men (n = 707)	*P*-value	Women^†^	Men^†^	*P*-value
**12-month**						
TWL, kg	32.5 ± 7.3	33.1 ± 7.4	0.150	32.0 (0.4)	32.6 (0.3)	0.181
BMIL, kg/m^2^	15.0 ± 4.3	15.3 ± 4.5	0.214	14.9 (0.2)	15.2 (0.2)	0.174
EBMIL, %	74.6 ± 18.6	75.6 ± 17.2	0.321	72.7 (0.8)	74.1 (0.8)	0.374
Δ Fat mass, kg	−29.6 ± 9.2	−37.0 ± 12.8	< 0.001	−29.2 (0.6)	−36.9 (0.7)	< 0.001
**24-month**						
TWL, kg	33.6 ± 8.3	33.4 ± 8.4	0.787	33.3 (0.6)	32.9 (0.6)	0.657
BMIL, kg/m^2^	15.6 ± 4.7	15.5 ± 5.0	0.929	15.6 (0.3)	15.5 (0.4)	0.815
EBMIL, %	76.6 ± 21.0	75.7 ± 19.0	0.582	75.5 (1.4)	74.0 (1.5)	0.432
Δ Fat mass, kg	−30.7 ± 9.8	−34.2 ± 13.2	0.032	−29.9 (1.2)	−34.3 (1.5)	0.013
**36-month**						
TWL, kg	30.6 ± 8.7	30.8 ± 9.1	0.876	30.2 (0.8)	30.7 (0.9)	0.653
BMIL, kg/m^2^	14.2 ± 4.9	13.9 ± 5.3	0.650	14.1 (0.5)	14.0 (0.5)	0.873
EBMIL, %	69.8 ± 20.3	69.1 ± 21.8	0.768	68.9 (1.9)	70.4 (2.2)	0.568
Δ Fat mass , kg	−24.3 8.5	−28.8 ± 16.5	0.136	−24.7 (2.5)	−29. (2.8)	0.227

TWL, total weight loss; BMIL, body mass index loss; EBMIL, excess body mass index loss.

*Model adjusted for preoperative diabetes mellitus, hypertension and dyslipidemia.

^**†**^Data are presented as mean (Standard Error).

Remission of diabetes mellitus, hypertension, and dyslipidemia after 12 postoperative months in men and women patients was 94.7% vs 93.1% (odds ratio [OR] = 2.33 [0.60–9.02], *P* = 0.220), 89.7% vs 94.3% (OR = 0.73 [0.29–1.81], *P* = 0.493), and 69.3% versus 77.2% (OR = 1.08 [0.51–2.29], *P* = 0.847), respectively.

The duration of operation was 113.5 ± 27 and 109.3 ± 26.9 min in men and women, respectively (*P* = 0.007); however, after adjustment for preoperative diabetes mellitus, hypertension, and dyslipidemia, there was no significant difference between the two sexes regarding the duration of operation (*P* = 0.147). No patient required conversion to open surgery. There was no difference between men and women regarding the length of postoperative hospital stay (2.2 ± 0.6 vs 2.3 ± 1.4 days in men and women, respectively; *P* = 0.196).

We observed no significant sex disparity in the incidence of postoperative in-hospital complications, with bleeding was the most common complication. There was also no significant difference between sexes in early (30-day) or late readmission or reoperation. As demonstrated in Table 4, the rate of in-hospital, 30-day and late complications was similar between men and women according to Clavien–Dindo classification. There was only one death in a male patient who underwent urgent peritoneal lavage and antimicrobial therapy following peritonitis after OAGB, but expired 28 days after surgery due to Pseudomonas Aeruginosa ventilator-associated pneumonia.

**Table 4:** Matched comparison of postoperative complications between men and women according to the Clavien–Dindo classification.

	Women (n = 707)	Men (n = 707)	Grade
**In-hospital, n**			
Vomiting	2	0	I
Bleeding	6	8	II
Hospital-acquired pneumonia	0	2	II
Other infection	2	1	II
Pulmonary embolism	1	3	IVa
Abscess due to leak	2	4	IVb
**30-day, n**			
Vomiting	3	2	II
Marginal ulcer	1	0	II
	0	1	IIIb
Bleeding	0	1	II
Other infection	0	3	II
Intestinal obstruction	2	0	IIIb
Gastroesophageal reflux	0	2	IIIa
Bleeding	5	3	IIIb
Abscess due to leak	3	5	IVb
Peritonitis	0	1	V
Other	0	2	II
**Late, n**			
Bleeding	0	1	II
Other	0	3	II
Intestinal obstruction	2	0	IIIb
Gastroesophageal reflux	1	1	IIIb
Abscess due to leak	1	1	IVb

Clavien–Dindo classification	Women	Men	*P*-value
Grade I	2	0	0.500
Grade II	12	23	0.060
Grade IIIa	0	2	0.500
Grade IIIb	10	5	0.194
Grade IVa	1	3	0.624
Grade IVb	6	10	0.315
Grade V	0	1	> 0.999
≥Grade I	31	44	0.123
≥Grade II	29	44	0.071
≥Grade III	17	21	0.511
≥Grade IV	7	14	0.124

## Discussion

In this matched-pair cohort analysis, bariatric surgery resulted in comparable short- and mid-term efficacy in men and women, with no significant differences in the incidence of postoperative in-hospital complications and early or late readmission and reoperation.

We observed no differences between men and women comparing weight reduction indices during 36 months postoperatively and 12-month remission of diabetes mellitus, hypertension and dyslipidemia. Parri et al. found that a younger age, a lower preoperative BMI, and RYGB versus SG, but not sex, were the independent predictors of 4-year weight loss after bariatric surgery^14^. Similarly, a recent study by Tankel et al. demonstrated that age, but neither sex nor preoperative BMI, could predict weight loss during seven years after surgery^15^. Benaiges et al. showed that the number of the anti-hypertension drugs taken prior to surgery and a slower weight loss, but not age or sex, were associated with lower hypertension remission rates after 12 months and a higher disease recurrence rate 36 months after surgery^16^. In a single-center study on 79 men and 79 women matched for age, preoperative BMI, diabetes mellitus, the type of bariatric procedure, and obstructive sleep apnea, no significant sex differences were noted comparing EBMIL%, blood pressure, and HbA1C reduction at 24-month postoperatively^17^. Finally, a matched-pair analysis by Tymitz et al. demonstrated similar BMIL values between men and women at 6- and 12-month after laparoscopic RYGB^18^.

In this study, we noted no sex differences regarding the length of postoperative hospital stay. In agreement with our observation, in a retrospective comparative study on 53 men and 106 women matched for preoperative BMI, age, as well as the preoperative prevalence of diabetes mellitus, hypertension, asthma, and sleep apnea, Tymitz et al. demonstrated that the length of hospital stay following laparoscopic RYGB was similar between the two sexes^18^. In our analysis, although the operation time was longer in men than in women, this difference was not significant after adjustment for preoperative comorbidities. Similarly, in the matched analysis conducted by Tymitz et al., the duration of laparoscopic RYGB was significantly longer in men than in women. In contrast; however, in a retrospective analysis on a large American database by Dugan et al., the longer durations of laparoscopic SG and RYGB in men than in women remained significant after adjusting for preoperative comorbidities, which was suggested by the authors to be in part attributable to thicker abdominal wall muscle and fascia and greater intra-abdominal adipose tissue in men^19^.

Men have been historically considered to be at higher risk for bariatric surgery compared to women, the perception of which is suggested to be a barrier to bariatric surgery in men. In our experience, male patients were significantly younger and presented with greater BMIs. Also, the prevalence of hypertension and dyslipidemia were higher in men than in women at the time of surgery. We observed no significant sex disparity in the incidence of postoperative in-hospital complications, early or late readmission or reoperation. However, a 10-year observation in the United States demonstrated that male sex was independently associated with a greater risk of in-hospital complications and mortality^20^; and using New York’s inpatient discharge database including 7868 patients who underwent bariatric surgery in 2003, Weller et al. identified male sex as an independent risk factor for postoperative in-hospital complications^21^. Nevertheless, the underlying mechanisms through which male sex influences postoperative in-hospital complications still remain nebulous and need to be elucidated in future investigations. We also observed that male sex was not associated with the increased risk of 30-day or late readmission and reoperation. In agreement, in a retrospective analysis of a national database in the United States (MBSAQIP) from 2015 to 2017, Dugan et al. showed that the higher incidence of postoperative complications in men than in women lost its significance after adjusting for preoperative comorbidities^19^. Also, a prospective observational study in Minnesota showed that prolonged hospital stay and open (versus laparoscopic) surgery, but not sex, were the independent predictors of 30-day readmission following primary RYGB^22^. In their retrospective matched analysis, Tymitz et al. observed no significant difference between the sexes regarding early and late complications and mortality^18^. Similarly, in a statewide study in New York, sex was not associated with the increased risk of 30-day readmission to the emergency department after the primary adjustable gastric banding, RYGB, and SG surgeries^23^.

There is a growing body of evidence indicating that men are generally less likely to seek healthcare services than women. This is thought to be in part a result of an interplay between psychosocial and cultural factors, causing sex differences in perceptions of body weight and obesity-related quality of life. This also makes men less motivated to pursue weight management strategies and request evaluation for surgery^24,25^. In this regard, patient referral patterns also seem to have a profound effect. It has been documented that patients are more likely to pursue bariatric surgery when it is recommended by their practitioners^26,27^. A systematic review has identified an association between female sex and greater willingness to undergo bariatric surgery and demonstrated that limited patients’ and practitioners’ levels of knowledge on bariatric surgery and their concerns about postoperative complications were major barriers to undergo surgery^26^. Nevertheless, according to the limited available guidelines developed by bariatric surgeons, the patient’s sex is not yet considered in decision-making for patient selection. Hence, a better understanding of the association of sex with the outcomes of bariatric surgery is helpful for practitioners, surgeons, and patients in setting realistic postoperative goals and expectations.

Including data from a well-defined continuous cohort and the use of a matched-pair design are the major strengths of the present study. Moreover, the data used for this study was from the patients operated by a single surgeon, which eliminates the risk of the inherent bias introduced by differences in surgeons’ skills or experiences. Nevertheless, this study has several limitations that deserve consideration. The first limitation is the retrospective nature of the study despite the fact that the data were collected prospectively. Moreover, despite a relatively large sample size, the present study may not still be able to provide strong evidence on differences of rare complications and mortality between the sexes because of the low incidence. Also, there were large proportions of missing data regarding postoperative comorbidities, which precludes any conclusion about possible differences in long-term disease recurrence. Finally, even with pairwise matching for multiple characteristics, the unavailability of data regarding socioeconomic factors, dietary habits, and physical activity behaviors of the participants may still have confounded our findings.

## Conclusion

Our matched-pair cohort analysis demonstrated that bariatric surgery results in comparable short- and mid-term efficacy in men and women, and is associated with similar rate and severity of postoperative complications between sexes. These findings suggest bariatric surgeons not to consider sex for patient selection in bariatric surgery. Further randomized clinical trials are warranted to verify our findings in the mid and long term after bariatric surgery.

## Data Availability

The datasets used and analyzed during the current study available from the corresponding author on reasonable request.
